# Small Bowel Obstruction due to Migrated Intragastric Balloon: A Case Report and Literature Review

**DOI:** 10.1155/2022/1440441

**Published:** 2022-12-30

**Authors:** Min Yien Tan, Kar Yin Fok, Huong Nguyen, Senarath Edirimanne, Michael Devadas

**Affiliations:** ^1^Department of General Surgery, Nepean Hospital, Kingswood, NSW 2747, Australia; ^2^Department of Upper Gastrointestinal Surgery, Nepean Hospital, Kingswood, NSW 2747, Australia

## Abstract

**Introduction:**

With the rising rate of obesity world-wide, there are increasing weight loss options including operative and non-operative techniques. Endoscopic intragastric balloons (IGB) have gained popularity since its inception three decades ago and is viewed as a less invasive alternative to bariatric surgery. However, complications, though rare and probably under-reported, can be associated with significant morbidity and mortality. *Case Presentation*. We present the case of a 44-year-old woman who presented with a two-day history of upper abdominal pain, nausea, and obstipation, on the background of a Spatz3™ Balloon (Spatz FGIA, Great Neck, NY, USA) endoscopically placed seven months prior. Computed tomography scan confirmed small bowel obstruction due to a migrated IGB, requiring laparotomy and enterotomy for retrieval.

**Conclusion:**

With the development of new types of IGB and increasing usage, it is important to monitor for issues and complications.

## 1. Introduction

The increasing prevalence of obesity is a world-wide health concern due to its association with many chronic, debilitating comorbidities. Bariatric surgery with maintained lifestyle changes is the most effective method to achieve and maintain weight loss; however, patients are often reluctant to pursue surgical intervention due to perceived risks or prohibitive costs. Hence, the development of bariatric endoscopy to deliver minimally invasive treatment has been a growing field since its conception in the 1980s [1]. Intragastric balloons (IGB) are endoscopically placed space-occupying device left implanted for six or twelve months to induce satiety and are generally considered low risk [2], although complications are likely under-reported. We present the case of a migrated IGB causing mechanical small bowel obstruction.

## 2. Case Presentation

A 45-year-old woman (initial weight: 103 kg; BMI 35.6 kg/m^2^) presented with two days of upper abdominal pain, nausea, and obstipation. She had a Spatz3™ Balloon (Spatz FGIA, Great Neck, NY, USA) placed endoscopically seven months prior, which has resulted in 7 kg of weight loss [% total body weight loss (TWL): 7%; % excess weight loss (EWL): 23%; change in BMI: 2.4 kg/m^2^]. She has not reported any previous abdominal discomfort nor noticed discoloration of her urine during this time, which might suggest a rather slow leakage from the IGB. There were no issues on initial follow-up with her bariatric surgeon, and she is due to follow-up again at the one-year mark for removal. The patient had no medical comorbidities and had not undergone previous abdominal surgery. On examination, her vital signs were normal. Her abdomen was distended but soft, with epigastric tenderness but no peritonism.

**Figure 1:**
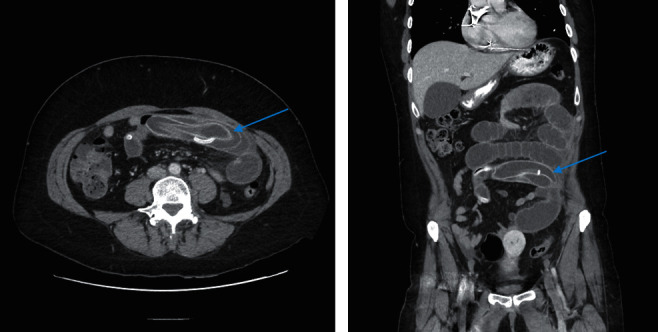
Computed tomography abdomen pelvis, portal venous phase showing small bowel obstruction due to migrated intragastric balloon (blue arrow). (a) Axial view. (b) Coronal view.

Computed tomography scan of the abdomen and pelvis showed small bowel obstruction with a transition point in the mid small bowel due to the IGB (Figures 1(a) and 1(b)). There was oedema of the proximal small bowel and mesentery, suggesting venous congestion, with a small amount of free fluid, but no free gas or pneumatosis.

**Figure 2:**
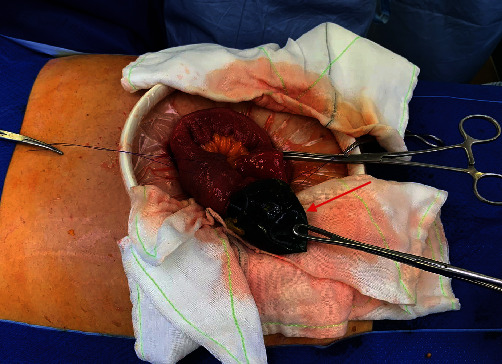
Intraoperative photo of IGB (red arrow) removed from small bowel via enterotomy performed.

Following fluid resuscitation and nasogastric tube insertion for decompression, she underwent a laparotomy that evening. Laparoscopy was not considered in this case due to several reasons, such as the difficulty of laparoscopic surgery with distended bowel loops and the risk of iatrogenic enterotomy in the context of a small bowel obstruction. The eventual conversion to laparotomy was also deemed necessary to safely extract the IGB, especially without the information regarding the type of IGB implanted at that point. Certain IGBs, such as Spatz ABS balloons, have an anchoring device, which may be difficult to remove laparoscopically [3]. Intraoperatively, the IGB was identified in the small bowel causing obstruction with proximal dilatation and distal collapsed small bowel loops. All bowel was viable, and there was no perforation or intraabdominal contamination. Enterotomy was created, and the balloon was milked back and retrieved in entirety (Figure 2). The enterotomy was closed transversely, and the rest of the small bowel run was normal. Laparotomy was closed with fascial sutures and skin clips. On inspection of the balloon, it was collapsed but has no obvious leakage point and still contained some saline with methylene blue.

Post-operatively, she had a period of ileus treated conservatively and superficial wound infection treated with antibiotics and wound packing. She remained well on follow-up three months post-operatively.

## 3. Discussion

The first IGBs inserted in 1985 were Garren Edwards gastric bubbles made of polyurethane and filled with 200–220 ml of air [1]. These were later withdrawn in 1988 due to the high number of adverse events from gastric erosion and spontaneous deflation leading to bowel obstruction. IGBs have since underwent structural remodeling and most IGBs now also contain methylene blue, so that blue/green urine can be used as a marker for deflation. There are currently around ten IGBs available on the market but only four U.S. Food and Drug Administration (FDA)-approved. This includes the IGB in this case that has most recently been approved in October 2021—the Spatz3™ Balloon (Spatz FGIA) [4]. The Spatz3™ Balloon has the benefit of a longer implantation time of 12 months and an adjustable volume post-implantation, which can improve tolerance [5].

IGB is a temporary weight control option, offered as an alternative therapy to bariatric surgery, or as an adjunct therapy prior to bariatric surgery. It is currently offered to patients between BMI 27 and 40 [2, 4] but has also been used in patients with BMI > 40 as a bridge to bariatric surgery. Patients, who are offered IGB as alternative therapy either, do not qualify for bariatric surgery based on the BMI cut-off, have high surgical risk, or simply prefer non-surgical management [2]. Its use as a bridging therapy to bariatric surgery especially in patients with severe obesity is postulated to be able to reduce perioperative morbidity, technical difficulty of the procedure, and operating time. However, the effects on post-operative complications and the ideal time to proceed with bariatric surgery requires further investigation [6].

**Figure 3:**
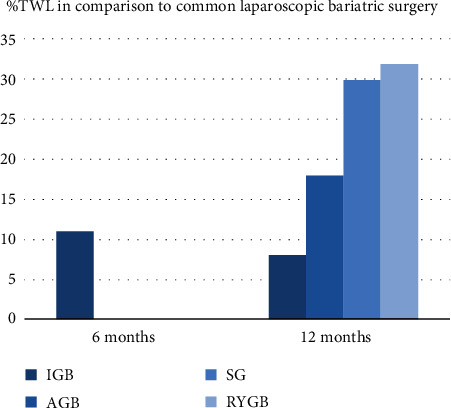
Comparison of weight loss efficacy (% total body weight loss) between IGB and common laparoscopic bariatric surgery at 6 and 12 months [7, 10, 11]. IGB: intragastric balloon; ABG: adjustable gastric band; SG: sleeve gastrectomy; RYGB: Roux-en-Y gastric bypass.

**Figure 4:**
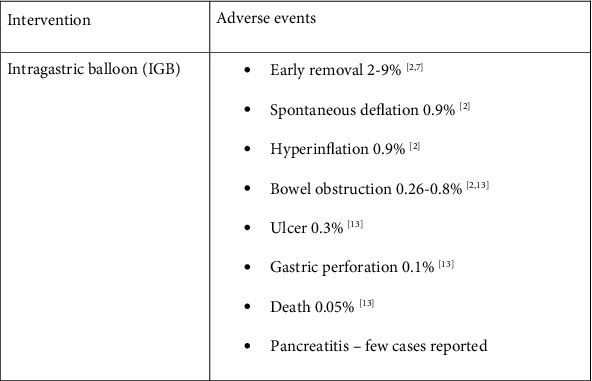
Side-effect profiles of IGB.

IGB offers an average total weight loss of around 7–17 kg (% TWL: 7–15%; % EWL: 22–50%) [7] at time of removal, with maintenance of 7–9 kg (8–9% TWL; 29–33% EWL) [7] TWL at 1 year. It also leads to improvement in metabolic syndrome, such as type 2 diabetes mellitus, hypertension, hypertriglyceridemia, and hepatic steatosis [8] with sustained results in a 1-year follow-up study [9]. However, as expected, its efficacy pales in comparison to other common laparoscopic bariatric surgeries, such as adjustable gastric band, sleeve gastrectomy, and Roux-en-Y gastric bypass (Figure 3) [7, 10, 11]. The most common side effects are abdominal pain, nausea and vomiting, and reflux [12]. This leads to early removal in 2–9% of patients [2, 7], but usually effectively resolves symptoms of intolerance with no longstanding sequelae. Severe complications are rare (Figure 4) [2, 12].

Spontaneous deflation occurs in 0.9% cases, but less than one third of these will migrate. Of the IGBs that do migrate, approximately one third (overall 0.06%) will require surgical intervention [2]. A review of the literature reveals 34 case reports in the last 35 years. They mostly occurred in IGB left in situ for longer than the indicated timeframe, but can also occur prematurely. The small bowel was the most common site of impaction presumably because the IGB is likely to pass spontaneously once it reaches the large bowel; however, unusual sites of obstruction, such as the sigmoid colon and Meckel's diverticulum, have been reported [13, 14]. Removal of the IGB was commonly via laparotomy or laparoscopy but can also be removed endoscopically or by spontaneous passage post percutaneous deflation of the balloon. Of the cases where laparoscopy was converted to laparotomy, risk of contamination, the patient's body habitus, and inability to access site of obstruction were reasons provided [14–16].

## 4. Conclusion

The efficacy of IGB in short-term weight reduction via a minimally invasive approach is an appealing attribute. As it continues to have an increasing uptake in the population, we have to be vigilant of potential complications. There should be a high level of suspicion of bowel obstruction when patients present with abdominal pain, nausea, and vomiting even though these symptoms are the most common side effects of IGB.
